# Expressed Prostate Secretions in the Study of Human Papillomavirus Epidemiology in the Male

**DOI:** 10.1371/journal.pone.0066630

**Published:** 2013-06-14

**Authors:** Vitaly Smelov, Carina Eklund, Davit Bzhalava, Andrey Novikov, Joakim Dillner

**Affiliations:** 1 Department of Laboratory Medicine, Karolinska Institutet, Stockholm, Sweden; 2 Department of Urology and Andrology, North-Western State Medical University named after I.I. Mechnikov, St. Petersburg, Russia; 3 St. Petersburg State University Outpatient Clinic, St. Petersburg, Russia; 4 Medical Center “Mikrobiomed” and Laboratory of Microbiology, D.O. Ott Research Institute of Obstetrics and Gynaecology, St. Petersburg, Russia; IPO, Inst Port Oncology, Portugal

## Abstract

**Introduction:**

Exploring different sampling sites and methods is of interest for studies of the epidemiology of HPV infections in the male. Expressed prostate secretions (EPS) are obtained during digital rectal examination (DRE), a daily routine urological diagnostic procedure, following massage of the prostate.

**Materials and Methods:**

Urethral swabs and EPS samples were obtained from a consecutive sample of 752 men (mean age 32.4 years; median life-time sex partners 34) visiting urology outpatient clinics in St. Petersburg, Russia and tested for HPV DNA by general primer PCR, followed by genotyping using Luminex.

**Results:**

Overall, 47.9% (360/752) of men were HPV-positive, with 42.0% (316/752) being positive for high-risk (HR-) HPV and 12.6% (95/752) for multiple HPV types. HPV-positivity in the EPS samples was 32.6% (27.7% HR-HPV) and in the urethral samples 25.9% (24.5% HR-HPV). 10.6% were HPV positive in both EPS and urethral samples. 6.4% had the same HPV-type in both EPS and urethral samples. 10.6% were HPV positive in both EPS and urethral samples. 6.4% had the same HPV-type in both EPS and urethral samples. The concordance between the urethral samples and EPS was 62.5% (470/752), with 80 cases double positive and 390 cases double negative in both sites. The sensitivity of urethral samples for overall HPV detection was 54.2% (195/360). Compared to analysis of urethral samples only, the analysis of EPS increased the HPV prevalence in this population with 26.2%.

**Conclusion:**

EPS represent informative sampling material for the study of HPV epidemiology in the male.

## Introduction

Studies of the epidemiology of Human Papillomavirus (HPV) infection in the male are essential for understanding HPV natural history, transmission dynamics and optimal uses of HPV vaccines. Research on optimal methods and anatomical sites for sampling are essential for enabling informative studies. A variety of different anatomical sites in men have been studied, including glans penis, prepuce, penile shaft, distal urethra, anal canal, perianal area, scrotum, semen and urine [1]–[5], but prostate secretions have to our knowledge not been studied so far. Expressed prostate secretion (EPS) is obtained during digital rectal examination (DRE, a daily routine urological diagnostic procedure) following massage of the prostate, and could thus represents a convenient new type of sample for studies of the epidemiology of HPV in the male.

EPS has been commonly used in urology ever since the Meares-Stamey 4-glass test, which included a quantitative culture of pure EPS, was launched in 1968 [6] and became the gold standard for assessing the bacteriological and inflammatory state of the lower urinary tract in urological patients. The two-glass or pre-post-massage screening test [7], [8] is recommended by European Association of Urology to differentiate between bacterial prostatitis and chronic pelvic pain syndrome in patients with prostatitis-like symptoms, if an acute urogenital infection and STD can be ruled out [9].

More than 155 different HPV types have been identified and are referred to by number [10]. Efficient genotyping can be achieved using bead-based multiplex genotyping [11] of PCR amplimers obtained following general primer PCR [12]. A modified general primer (MGP) system was established as a modification of the classical HPV general primer system GP5+/6+ and found to result in an improved and more uniform analytical sensitivity for different HPV types [12]. The system has also been validated using the WHO HPV DNA genotyping proficiency panel [13], [14].

Using the multiplexed genotyping method, we explored whether HPV was detectable in EPS and whether analysis of EPS would yield any additional information compared to analysis of samples from the distal urethra.

## Materials and Methods

### Study design

A consecutive series of men who were visiting for STD testing in the urology units of two university outpatient clinics and a large clinic that provides testing for HIV and STDs to the general population in St. Petersburg were invited to participate in the study from May 2006 to February 2009. A total of 760 men, aged 18–60 (mean age 31.7) years, were enrolled after receiving a written instruction and providing written informed consent. The institutional review board of St. Petersburg State University and Medical Academy of Postgraduate Studies (North-Western State Medical University named after I.I. Mechnikov since 2011) approved the study.

### Procedure

A urologist obtained a detailed medical history and performed a physical examination, including DRE. We also collected data on lifestyle and sexual behaviors, such as age at first intercourse, number of lifetime sex partners, sex preferences, and STDs history. All men provided blood samples for HIV and *Treponema pallidum* testing. In men over 40 years, prostate-specific antigen (PSA) testing was also performed.

Before sampling, men were instructed to abstain from any form of sex for 3–5 days and from urination for 3–4 hours. The study clinician sampled the urethra with a urethral swab. Once inserted into the urethra, the swab was rotated 180° right- and leftwards. Then the swab was rinsed in 1000 µl of phosphate buffer in two separate tubes. The first sample was used for detection for STDs, such as *Chlamydia trachomatis*, *Neisseria gonorrhea*, *Trichomonas vaginalis*, and herpes simplex virus (HSV) types 1 and 2 at the local laboratory, while the second sample was stored at −20°C prior to HPV testing. After urethral sampling, the man was asked to urinate. Afterwards a DRE with massage of the prostate was performed. The EPS was allowed to drop freely out of the urethra directly into a separate tube, which was also stored at −20°C prior to HPV testing.

### Detection and typing of HPV

HPV testing of urethral and EPS samples was conducted using PCR amplification of a fragment in the L1 gene [12]. DNA was released using a freeze-thaw-boil procedure, as described [15]. In brief, 100 µl the sample was centrifuged and reconstituted in 100 µl of TE-buffer pH 7.5. This sample was boiled at 107°C degrees for 10 minutes, after which it was frozen at −20°C degrees. The quality of the sample DNA for amplification was analysed using a β-globin real-time PCR using 5 µl of the sample in a 25 µl reaction [16]. Samples testing β-globin negative were treated with SDS/Proteinase K over night as described [16] and the real-time PCR repeated. β-globin was detected in 98.9% (1504/1520) of the urogenital samples. Eleven still β-globin negative samples (3 urethral and 8 prostate, 8 men in total) were excluded from the present study. Complete urethral and prostate paired sample sets could thus be obtained from 752 men. Samples were tested for the presence of HPV by amplifying 5 µl of DNA with the MGP primer system, as described [12]. HPV detection and genotyping was conducted on the Bioplex 200 Luminex system (Bio-Rad, Hercules, CA, USA) using multiplex bead-based hybridization with Luminex technology as described by Schmitt et al [11]. In brief, 10 µl of the biotinylated MGP-PCR products were mixed with beads coupled with different HPV probes, after 10 minutes denaturation at 95°C the samples were incubated at 41°C for 30 minutes. After washing, streptavidin-R-phycoerythrin was incubated with the samples for 30 minutes at room temperature. 100 beads of each HPV type from each sample were analysed using the Luminex system. The probes for 14 oncogenic (high-risk, HR-HPV) types (16, 18, 31, 33, 35, 39, 45, 51, 52, 56, 58, 59, 66, 68a and 68b, including probes for variant sequences of HPV-18, 35, 51 and 58) and 22 non-oncogenic types including low-risk and possible high-risk types (6, 11, 26, 30, 40, 42, 43, 53, 54, 61, 67, 70, 73, 74, 81, 82, 83, 86, 87, 89, 90 and 91) were used. Eleven negative controls (H_2_O) and 8 positive controls (HPV plasmid pools) were run on every PCR and Luminex plate.

### Statistical analysis

HPV and HR-HPV concordance was calculated as the proportion of men double positive or double negative for both urethra and prostate specimens as compared to the full cohort. Comparisons of HPV prevalences and determinants of HPV infection used binomial logistic regression to calculate odd-ratios (ORs) and their confidence intervals (95%CI) using the statistical software R (R Development, Core, Team. R: A language and environment for statistical computing. R Foundation for Statistical Computing. 2010; Available from: http://www.R-project.org).

## Results

Among the 752 men tested, the HPV prevalences were 25.9% (195/752) in the urethral samples and 32.6% (245/752) in the EPS samples. Oncogenic HPV infections (HR-HPV) were found in 24.5% (184/752) and 27.7% (208/752) of the urethral and EPS samples, respectively.

Combining urethral and EPS samples resulted in an overall 47.9% HPV-positivity in the studied population, twice as much as seen when analyzing only samples from the distal urethra. Similarly, the combined prevalence for oncogenic (high-risk, HR-HPV) and multiple HPV infection was 42.0% and 12.6%, respectively (Table 1).

**Table 1:** Detection of human papillomavirus (HPV) infection in the male distal urethra as compared to in expressed prostate secretions.

HPV types	Urethra (%)	Prostate (%)	Urethra or Prostate (%)	Urethra and Prostate (%)
6	16 (2.1)	8 (1.1)	24 (3.2)	0
11	2 (0.3)	4 (0.5)	6 (0.8)	0
16	67 (8.9)	81 (10.8)	129 (17.2)	19 (2.5)
18	24 (3.2)	10 (1.3)	31 (4.1)	3 (0.4)
26	0	0	0	0
30	4 (0.5)	6 (0.8)	10 (1.3)	0
31	1 (0.1)	3 (0.4)	3 (0.4)	1 (0.1)
33	2 (0.3)	1 (0.1)	3 (0.4)	0
35	3 (0.4)	7 (0.9)	10 (1.3)	0
39	0	3 (0.4)	3 (0.4)	0
40	1 (0.1)	5 (0.7)	6 (0.8)	0
42	8 (1.1)	9 (1.2)	15 (1.9)	2 (0.3)
43	6 (0.8)	5 (0.7)	11 (1.5)	0
45	21 (2.8)	20 (2.7)	35 (4.4)	6 (0.7)
51	17 (2.3)	9 (1.2)	25 (3.3)	1 (0.1)
52	5 (0.7)	9 (1.2)	13 (1.7)	1 (0.1)
53	3 (0.4)	5 (0.7)	6 (0.8)	2 (0.3)
54	0	0	0	0
56	2 (0.3)	6 (0.8)	8 (1.1)	0
58	2 (0.3)	5 (0.7)	6 (0.8)	1 (0.1)
59	11 (1.5)	7 (0.9)	16 (2.1)	2 (0.3)
61	0	0	0	0
66	11 (1.5)	31 (4.1)	38 (5.1)	4 (0.5)
67	4 (0.5)	6 (0.8)	9 (1.2)	1 (0.1)
68	0	1 (0.1)	1 (0.1)	0
70	1 (0.1)	5 (0.7)	6 (0.8)	0
73	1 (0.1)	0	1 (0.1)	0
74	5 (0.7)	12 (1.6)	17 (2.3)	0
81	3 (0.4)	3 (0.4)	6 (0.8)	0
82	2 (0.3)	1 (0.1)	3 (0.4)	0
83	0	1 (0.1)	1 (0.1)	0
86	1 (0.1)	2 (0.3)	3 (0.4)	0
87	10 (1.3)	21 (2.8)	29 (3.9)	2 (0.3)
89	0	3 (0.4)	3 (0.4)	0
90	2 (0.3)	7 (0.9)	9 (1.2)	0
91	8 (1.1)	16 (2.1)	21 (2.8)	3 (0.4)
HPV+ men, in total	195 (25.9)	245 (32.6)	360 (47.9)	80 (10.6)
HPV- men, in total	557 (74.1)	507 (67.4)	674 (89.6)	390 (51.9)
HR-HPV+ men	184 (24.5)	208 (27.7)	316 (42.0)	76 (10.1)
LR-HPV+ men	81 (10.8)	125 (16.6)	189 (25.1)	17 (2.3)
Single HPV infection	156 (20.8)	184 (24.5)	296 (39.4)	44 (4.9)
Multiple HPV infection	39 (5.2)	61 (8.1)	95 (12.6)	5 (0.7)
No. studied men, total	**752**			

The 5 most common HPV types were HPV-16 (17.2%), HPV-66 (5.1%), HPV-45 (4.4%), HPV-87 (3.9%) and HPV-6 (3.2%). In the male distal urethra the 5 most common HPV types were HPV-16 (8.9%), followed by HPV-18, -45, -51 and -6 (3.2%, 2.8%, 2.3% and 2,1%), whereas the 5 most common types were HPV-16 (10.8%), followed by HPV-66, -87, -45 and -91 (4.1%, 3.9%, 2.7% and 2.1%) in the prostate secretion specimens. Three of the HPV types tested for were not detected in any sample (HPV-26, -54 and -61). Single HPV infections were found in 20.8% (n = 156) of urethral and 24.5% (n = 184) of prostate samples but only 44 men (4.9%) were positive for the same single HPV type in both types of samples (Table 1). Multiple HPV infections were common, found in 12.0% (n = 90) of the men. Multiple HPV infections were less common in the distal urethra (5.2%) than in the prostate secretion (8.1%) specimens (p = 0.002). Up to 5 concomitant HPV infections were seen in urethral samples and up to 4 in the EPS. Up to 3 concomitant HR-HPV types could be present in both sites.

HR-HPV infection tended to be more common and LR-HPV infection was significantly more common in the EPS than in the urethral samples (p = 0.06 and p = 0.0003, respectively).

HPV concordance between the distal urethra and prostate secretions specimens obtained from the same men was low: 10.6% (n = 80) for all HPV, 10.1% (n = 76) for HR-HPV and 2.3% (n = 17) for LR-HPV types, respectively, with only 6.4% (n = 48) and 5.1% (n = 38) being concordant based on exactly the same HPV or HR-HPV type, respectively. The sensitivity of urethral samples for overall HPV detection was 54.2% (195/360).

We found only one determinant of the HPV prevalences, namely a lower HPV prevalence with increasing age. This was found both for urethral (p = 0.009) and EPS (p = 0.003) samples. HPV prevalence was not significantly related to the life-time number of sexual partners (Table 2).

**Table 2:** Factors associated with detection of human papillomavirus (HPV) in the male urethra and expressed prostate secretions (EPS): Multivariate Analysis.

Factor		Urethra			EPS		
		Number of HPV+ men/total tested	OR (95%CI)	P-value	Number of HPV+ men/total tested	OR (95%CI)	P-value
Age at the study time, years	18–24	43/140	**Ref**		39/140	**Ref**	
	25–29	53/187	0.3 (0.1–0.93)	0.04	66/187	0.26 (0.09–0.75)	0.01
	30–34	48/190	0.32 (0.12–0.84)	0.02	60/190	0.33 (0.14–0.81)	0.02
	35–39	27/107	0.73 (0.24–2.24)	0.59	39/107	0.52 (0.18–1.53)	0.24
	40+	31/117	0.05 (0.01–0.48)	0.009	37/117	0.08 (0.02–0.43)	0.003
Age when sex life began, years	<16	42/135	**Ref**		53/135	**Ref**	
	16–19	128/464	1.18 (0.42–3.29)	0.77	136/464	1.31 (0.51–3.51)	0.58
	20+	31/141	2.95 (0.75–11.55)	0.12	53/141	2.37 (0.66–8.53)	0.19
Number of life-time sex partners	0–5	36/151	**Ref**		44/151	**Ref**	
	6–10	45/180	0.7 (0.21–2.35)	0.57	48/180	0.91 (0.27–2.88)	0.87
	11–20	40/150	1.0 (0.3–3.31)	1.0	52/150	1.21 (0.38–3.83)	0.74
	21+	81/259	1.04 (0.32–3.32)	0.95	95/259	1.49 (0.49–4.54)	0.48
Any STDs present	Not detected	227/709	**Ref**		256/709	**Ref**	
	Detected	14/47	1.45 (0.63–3.36)	0.38	20/47	1.3 (0.59–2.88)	0.52
History of treated Chlamydia infection	Never	126/504	**Ref**		156/504	**Ref**	
	Reported	76/237	1.06 (0.44–2.54)	0.89	86/237	0.99 (0.44–2.23)	0.99
Pain syndrome	No	146/506	**Ref**		181/506	**Ref**	
	Yes	59/212	0.82 (0.38–1.79)	0.63	64/212	0.81 (0.39–1.67)	0.57

Genital warts were found in 7 (0.9%) men in total; all of them were visibly presented on penile heads. The urethral swabs were negative for HPV infections for 5 of these men and 2 of the EPS samples were also negative.

Recently diagnosed STDs were present in 47 (6.3%) males: HIV in one (0.1%), HSV in 3 (0.4%) and *C. trachomatis* in 43 (5.7%) men with one (0.1%) positive case for *N. gonorrhea* among them. The HIV-positive man had multiple HPV infections (HPV types -16, -30, -33, -42 and -45 in the urethra and HPV-42 in the EPS). In the single case of gonococcal urethritis, HPV-91 was detected in both samples. Treatment against *C. trachomatis* infection in the past was reported by 31.5% (237) of the men. Only 10 (1.3%) men reported practicing sex with other men. There were no differences in HPV prevalences in relation to STD history, neither for urethral nor for prostate specimens (Table 2).

## Discussion

Prostate secretions were found to represent an informative sample material for the study of HPV epidemiology in the male, greatly increasing the number of HPV-positive cases as compared to the study of samples from the distal urethra. The concomitant study of EPS and urethral swabs was necessary in order to answer the question whether virus presence in the EPS may be derived from the urethra when the EPS is transported through the urethra. To minimize urethral contamination of the EPS samples, the men were asked to urinate between collecting samples, as is the recommended procedure for urological patients [7], [8]. As many EPS samples contained HPV also when the urethral samples were negative and as the HPV type-concordance was limited, most of the presence of HPV in the EPS samples is unlikely to be derived from the distal urethra. Although the prostate epithelium thus seems like the most likely source of the HPV found in the EPS, we cannot exclude that it may derive from adjacent anatomical structures, such as the seminal vesicles.

Semen samples have shown only low HPV prevalences [3], although EPS is a part of the ejaculate. Possible reasons for this discrepancy include different study populations, as a very high sexual risk taking population would be more likely to be HPV-positive and/or that EPS may be more diluted in semen. Compared to semen, EPS is more suitable for epidemiological studies as it is readily obtained during a single, routine visit.

This is the first and only study where multiplex high-throughput genotyping of an extended spectrum of HPV types was performed on prostate secretion samples, analyzed and compared with the paired distal urethra samples obtained from the same men, representing the male upper and lower urogenital tract, respectively.

A limitation of our study was that we did not compare the HPV prevalences in EPS to the HPV prevalences in samples from various parts of the body, such as penile or anal swabs. Penile swabs are reported to be the anatomic site with the highest published prevalence of HPV [17]. The aim of our study was merely to explore whether HPV would be commonly present in EPS and, if so, whether the presence of HPV in EPS could be explained by contamination from the distal urethra. Although our data indicate that EPS is an informative sample that should be considered for inclusion in comprehensive studies of HPV epidemiology of the male, the question of how it compares to all other possible samples and which panel of samples to include will need to be answered by future studies.

Prostate cancer is the most common non-cutaneous cancer among males [18]. Chronic inflammation of the prostate and history of STDs are among the factors speculated to be part of prostate carcinogenesis [19]–[25]. A possible involvement of HPV was suggested by a seroepidemiological study [26], but different studies found inconsistent associations with different HPV types and the original association was attributed to chance, bias, or confounding by some unknown risk factor that may associate with different HPV infections in different populations [27].

Studies of HPV DNA detection in prostate cancer tissue have been controversial. Although some studies have found HPV DNA [28]–[30] others did not [31]–[33]. In the current study, HPV DNA in EPS samples was found to be less common among men above 40 years of age. Absence of HPV DNA in prostate cancers, which occur among the elderly, does therefore not contradict the possibility that HPV may infect prostatic epithelium among younger, sexually active men.

A high number of female sex partners is a major risk factor for male acquisition of genital HPV infection [34]–[36], but this was not detected in the present study. A possible explanation is that the studied cohort had such a high number of sexual partners that also the reference category of men (who had less than 6 life-time partners) was already highly exposed.

HPV infection in men is often asymptomatic, resulting in a large number of asymptomatic carriers [37], and can clear spontaneously. HPV infection rates in males have been reported to range from 1.3% to 72.9%, with prevalences being dependent on the sampling and processing methods as well as the anatomic sites sampled [1], [17]. Penile and urethral brushings have been recommended as the most accurate method (or penile brushing and semen as an alternative) for HPV DNA detection in men [2]. However, that study was based only on 50 subjects [2]. A larger study on PCR detection of HPV in the urethra, different parts of penis, scrotum, perianal area and anal canal, semen and urine in 463 men found an overall HPV prevalence of 65.4%. Sampling from the penile shaft, glans penis/coronal sulcus and scrotum in heterosexual men was recommended [3]. In our study of 752 men, the HPV prevalences were 25.9% in the male distal urethra and 32.6% in the prostate secretion specimens resulting in an overall 47.9% of HPV-positivity. Because deep urethral sample collection is painful, selection of a convenient combination of samples that would still be able to detect most of the HPV infections in the study subjects is a challenge [3]. EPS tested alone or in a combination with urethral swabs might not provide the complete picture of overall HPV prevalence in a male, but it will broaden our knowledge on HPV epidemiology in the male urogenital tract, especially in some targeted populations (urological patients, elderly men, vaccinated people etc.) and, because of the presence of EPS in the ejaculate, the study of EPS is of particular interest in studies of the transmission of HPV to the sexual partner(s). Other studies where EPS might be of interest are to improve the diagnostics of HPV infection in men, studies monitoring the effect of HPV vaccination and studies of HPV infection in men with and without inflammatory processes in the prostate.

In summary, we find that expressed prostate secretions are commonly positive for HPV infections that are not detectable in urethral swabs. Continued studies using EPS might allow for a better understanding of HPV epidemiology and natural history, its involvement in the urological diseases in the male as well as its transmission to the sexual partner.
